# Associations between Multiple Accelerometry-Assessed Physical Activity Parameters and Selected Health Outcomes in Elderly People – Results from the KORA-Age Study

**DOI:** 10.1371/journal.pone.0111206

**Published:** 2014-11-05

**Authors:** Sandra Ortlieb, Lukas Gorzelniak, Dennis Nowak, Ralf Strobl, Eva Grill, Barbara Thorand, Annette Peters, Klaus A. Kuhn, Stefan Karrasch, Alexander Horsch, Holger Schulz

**Affiliations:** 1 Institute of Epidemiology I, Helmholtz Zentrum München, German Research Center for Environmental Health, Neuherberg, Germany; 2 Institute of Medical Statistics and Epidemiology, Technische Universität München, Munich, Germany; 3 Institute and Outpatient Clinic for Occupational, Social and Environmental Medicine, Ludwig-Maximilians-Universität, Munich, Germany; 4 Comprehensive Pneumology Center Munich (CPC-M), Member of the German Center for Lung Research, Munich, Germany; 5 Institute for Medical Information Processing, Biometrics and Epidemiology, Ludwig-Maximilians-Universität München, Munich, Germany; 6 German Center for Vertigo and Balance Disorders, Ludwig-Maximilians-Universität München, Munich, Germany; 7 Institute of Epidemiology II, Helmholtz Zentrum München, German Research Center for Environmental Health, Neuherberg, Germany; 8 Institute and Outpatient Clinic for Occupational, Social and Environmental Medicine, Ludwig-Maximilians-Universität, Munich, Germany; 9 Institute of General Practice, University Hospital Klinikum rechts der Isar, Technische Universität München, Munich, Germany; 10 Department of Computer Science, University of Tromsø, Tromsø, Norway; 11 Department of Clinical Medicine, University of Tromsø, Tromsø, Norway; University of Zaragoza, Spain

## Abstract

**Introduction:**

Accelerometry is an important method for extending our knowledge about intensity, duration, frequency and patterns of physical activity needed to promote health. This study has used accelerometry to detect associations between intensity levels and related activity patterns with multimorbidity and disability. Moreover, the proportion of people meeting the physical activity recommendations for older people was assessed.

**Methods:**

Physical activity was measured in 168 subjects (78 males; 65–89 years of age), using triaxial GT3X accelerometers for ten consecutive days. The associations between physical activity parameters and multimorbidity or disability was examined using multiple logistic regression models, which were adjusted for gender, age, education, smoking, alcohol consumption, lung function, nutrition and multimorbidity or disability.

**Results:**

35.7% of the participants met the physical activity recommendations of at least 150 minutes of moderate to vigorous activity per week. Only 11.9% reached these 150 minutes, when only bouts of at least 10 minutes were counted. Differences in moderate to vigorous activity between people with and without multimorbidity or disability were more obvious when shorter bouts instead of only longer bouts were included. Univariate analyses showed an inverse relationship between physical activity and multimorbidity or disability for light and moderate to vigorous physical activity. A higher proportion of long activity bouts spent sedentarily was associated with higher risk for multimorbidity, whereas a high proportion of long bouts in light activity seemed to prevent disability. After adjustment for covariates, there were no significant associations, anymore.

**Conclusions:**

The accumulated time in moderate to vigorous physical activity seems to have a stronger relationship with health and functioning when shorter activity bouts and not only longer bouts were counted. We could not detect an association of the intensity levels or activity patterns with multimorbidity or disability in elderly people after adjustment for covariates.

## Introduction

Population aging will be a substantial societal phenomenon in the next half century. Due to the increase in life expectancy and the associated increase in the number of individuals at risk for chronic diseases and injuries, the aging of the population will produce crucial societal challenges [1]. Physical activity (PA) is a fundamental component targeted at preventing diseases and maintaining functional independence as well as in therapeutic intervention and rehabilitation programs for older adults.

Many studies have shown significant associations between PA and certain chronic diseases, including coexistence of selected diseases with high prevalence (e.g. cardiovascular diseases and diabetes) [2], [3]. In contrast, studies about the associations of multimorbidity (the coexistence of multiple chronic diseases) and PA are rare. Only one article about this subject was included in the review by Marengoni et al. [4] in 2011, who examined occurrence, causes, and consequences of multimorbidity. Hudon et al. [5] did not identify an association between multimorbidity and PA. Recently, two further studies were published which showed an inverse association between PA and multimorbidity [6], [7]. The relationship between PA and functional limitations or disability has been better investigated: two reviews dealt with this topic and reported consistent results demonstrating a positive influence of PA on the relationship of aging and disability [8], [9]. However, all studies on the associations between disability or multimorbidity and PA used self-reports to assess PA.

Although moderate to vigorous physical activity (MVPA) has been shown to provide beneficial effects to the health and functioning of older people [10], [11], little is known about the impact of light PA and the activity patterns needed to promote health in elderly people. The global recommendations on PA from the World Health Organization (WHO) [11] suggest that adults, including older adults, should perform a minimum of 150 min of at least moderate intensity PA per week for beneficial health effects. Moreover, the activity should be performed in bouts of at least 10 min duration. Moderate PA is defined as considerable increases in heart rate and breathing, such as brisk walking, and vigorous PA is even more exhausting [12]. The proportion of elderly people who meet the recommended PA guidelines diminishes from 47% −63%, found through questionnaires, to 6% −26%, when assessed by use of accelerometers [13].

Due to low costs and high feasibility, PA was usually measured by means of questionnaires in large epidemiological studies. The instrument requires a thorough and objective consideration of the questions addressed but it often comes along with recall bias, socially desirable responses, and the influence of mood, depression, anxiety, cognition, and disability on responses [14]. Accelerometers resolve some of the limitations of self-report instruments: they are not affected by random and systematic errors introduced by respondents and interviewers, and they provide valid and reliable estimates about basic characteristics (frequency, duration and intensity of PA) as well as of PA patterns [15], [16]. Moreover, accelerometers have improved the ability to examine the relationship between PA and different health outcomes and thus present supportive statistics for public health planning and intervention [17].

Recently, sedentary PA, as characterized by activities involving mainly sitting, has been proposed as a risk factor for all-cause disease that is independent of MVPA [18]. The role of light PA (e.g. self-care, cooking, casual walking or shopping), measured by accelerometers, with regard to health effects has been less studied [19], [20]. This might be of particular interest for older people who are limited in their exercise capacity due to age-related physical or mental restrictions [21]. In this respect, the patterns of PA have been proposed as a new group of PA outcomes that may offer additional information beyond reports of activity counts and activity type recognition [22].

In our study, we will examine the associations between the volume of PA (divided into sedentary PA: ≤100 counts per min (cpm), light PA: 101–1951 cpm, and MVPA: ≥1952 cpm) as well as PA patterns (with the so called GINI-Index [22], [23] as quantification) measured by accelerometers and specific health outcomes (disability and multimorbidity). Each health outcome is a clinically distinct entity with different prognosis and health care implications [24] and should therefore be observed individually. In this context, we will answer the following research questions: 1) Is there an association between the volume of sedentary or light PA and the two health outcomes, independent of MVPA? 2) Is there an association between PA patterns and the two health outcomes? 3) Are there differences and similarities regarding the relationship of PA with disability and multimorbidity? The answers to these questions will allow us to present novel information about the associations between sedentary PA, light PA as well as PA patterns and different health outcomes. Furthermore, we will assess the proportion of people fulfilling the current global recommendations of PA for older people under different methodical conditions, i.e. assess the impact of different bout lengths varying between 1 and the WHO recommended 10 min in subjects with and without disability or multimorbidity, and translate our results into advice for the prevention of chronic diseases and disability in elderly people.

## Methods

The KORA-Age study was approved by the Ethical Committee of the Bavarian medical association (Ethik-Kommission Nr. 08064), written informed consent has been obtained from the participants and all investigations have been conducted according to the principles expressed in the Declaration of Helsinki.

### Study population

Data from the present study derived from the KORA (Cooperative Health Research in the Region of Augsburg)-Age cohort. The KORA-Age study investigates the determinants and consequences of health status changes of older adults in a representative population based sample over a period of 3 years. Details have been described previously [25]. Since lung function is an important predictor of morbidity and mortality [26], 200 eligible individuals with extreme lung function values within the normal range had been selected from the first and fourth quartiles of the study population and grouped into a ‘better’ and a ‘worse’ lung function group. Activity counts from the non-dominant side of the hip were measured in 191 subjects at a rate of 30 Hz and stored at an epoch length of 2 sec by means of a GT3X (ActiGraph, Pensacola, FL, USA) accelerometers during daily living. Data filtering was set to default (‘normal’) as recommended by ActiGraph. PA was recorded up to 10 days, starting from the day at the study center where the accelerometers were handed out, until the eleventh day, which was the day the subjects were instructed to return the devices. PA data from the first recorded day as well as any other day with a wear time of less than ten hours were excluded. To be included in the study, subjects had to have a minimum of four valid days. After exclusion of subjects with insufficient days of recordings (n = 19) or accelerometer malfunctions (n = 4), the final study sample comprised 78 men and 90 women. Participants had 8.1±1.5 (mean ± SD) days of valid accelerometer data. The mean wear time was 740±114 min per day.

Details about the study design, participants and other methodic procedures have been described previously [22].

### Measures

#### Independent variables

The independent variables included: gender; age (65–69, 70–74, 75–79, >79); BMI (under-weight if <18.5 kg/m^2^, normal weight if 18.5–24.9 kg/m^2^, overweight if 25.0–29.9 kg/m^2^ and obesity if ≥30.0 kg/m^2^); education (≤10 years vs. >10 years); smoking habits (never, formerly smoked vs. currently smokes); alcohol consumption (teetotalism vs. no teetotalism); lung function (‘better’ vs. ‘worse’ lung function group) and risk of malnutrition measured by the Geriatric Nutritional Risk Index (GNRI) (major risk if <82, moderate risk if ≥82 and <92), low risk if >92 and ≤98, and no risk if >98) [27]. No person was at major risk and only 4 people were on moderate risk. Therefore we merged moderate and low risk.

*Physical activity*: To represent the characteristics of PA, multiple variables were obtained from uniaxial accelerometer data. As an overall measure of PA average counts per minute (cpm) were calculated for each subject. Times in different intensity levels were calculated by the most commonly used PA cut points applied for adults: *sedentary PA* was defined as ≤100 cpm [14] and the cut points by Freedson et al. [28] were used for *light* (101−1951 cpm), *moderate* (1952−5724 cpm), and *vigorous PA* (5725−9498 cpm). *PA patterns:* In the present study, a *bout* is defined as consecutive min spent in a specific intensity level, i.e. sedentary PA, light PA or MVPA, without interruption. To determine the time participants spent in the MVPA level and the portion of people fulfilling the current PA recommendations of ≥150 min of MVPA per week [11], we applied different cut offs for the bout lengths, ranging from 1 min to 10 min: i.e. the recommended ≥150 min of MVPA per week had to be performed in bouts of at least 1 min up to at least 10 min. We averaged MVPA over all valid days of recordings and multiplied it by seven, in order to test if a person met the PA recommendations of ≥150 min per week.

Activity patterns are characterized by the distribution of frequency of bouts of different duration. This information is combined by the so-called *GINI-index (G)*, introduced by Chastin and colleagues [23]. The index expresses how the PA time in a specific intensity level is accumulated with respect to the bout lengths. The index value *G* ranges from 0 to 1 and has to be interpreted as follows: *High G values*: large difference between minimal and maximal bout lengths and relatively high proportion of few long bouts in relation to the overall time. *Low G values:* small difference between minimal and maximal bout length, i.e. the activity pattern is characterized by a lot of relatively short bouts of similar length. A G value of 0 would result if all the bouts were the same length. Note that this does not depend on the length of these bouts, but only on their relationship to each other.

#### Dependent variables

*Multimorbidity* was defined as the presence of ≥2 *chronic diseases* out of a list of 13 chronic diseases: hypertension, eye disease, heart disease, diabetes mellitus, joint disease, lung disease, gastrointestinal disease, mental disease, stroke, cancer, kidney disease, neurological disease, liver disease. Chronic health conditions were determined through a self-administered questionnaire and a standardized telephone interview adapted from the self-report-generated Charlson Comorbidity Index [29].

*Disability* was quantified using the Health Assessment Questionnaire Disability Index (HAQ-DI) [30] during a telephone interview. The instrument consists of 20 questions in eight domains (dressing and grooming, hygiene, arising, reach, eating, grip, walking, and common daily activities), which can be answered on a scale from 0 (no difficulty), 1 (some difficulty), 2 (much difficulty) to 3 (unable to perform). The HAQ-DI score is the mean of the eight domains. In line with the literature [31], disability was defined as HAQ-DI >0. The HAQ had shown high validity, a good test-retest reliability, and internal consistency, and can be applied very well to elderly people [31]. For more detailed information regarding disability within the KORA-Age framework see Strobl et al. [32].

### Statistical analyses

Some of the metric variables were not normally distributed. Thus, the Wilcoxon two-sample test was used for metric variables to test for differences between people with and without a diagnosis of multimorbidity or disability, and Chi^2^-test for categorical variables. For the multiple logistic regression models, we used the interquartile range (IQR) of each PA variable as scaling distance. The IQR is defined as the distance between the 25^th^ and 75^th^ percentiles and thus describes values of the predictor that are relatively well-represented in the sample [33]. The association between the six PA parameters (sedentary PA, light PA, MVPA, G for sedentary PA (G_sedentary_), G for light PA (G_light_) or G for MVPA (G_MVPA_)) and multimorbidity as well as disability was examined using multiple logistic regression models. Statistical models were adjusted for gender, age, BMI, education, smoking, alcohol consumption, lung function group, nutrition, and one of the health outcomes (multimorbidity, disability), as appropriate. Results are presented as odds ratios (OR) and 95% confidence intervals. Statistically significant differences were assumed at a significance level of p<0.05. Statistical analyses were conducted using SAS version 9.2 (SAS institute Cary, NC).

## Results

### Descriptive characteristics

Table 1 presents the characteristics of the study sample: 168 people (46.4% male, 53.6% female) were included in the study with a median (5%, 95%) age of 73 (65, 86) years and a median BMI of 27 (23, 35) kg/m^2^. More than half of the study sample (51.8%) was multimorbid and 41.7% of the participants were disabled. 29.7% of all participants were both disabled and multimorbid (results not shown in the table). More detailed information about the subject characteristics and clinical parameters stratified by multimorbidity and disability are presented in table 1 (median values) and table S1 (mean values).

**Table 1:** Characteristics of the participants, stratified by multimorbidity and disability. Median (5%, 95%).

	all	multimorbidity		disability	
	n = 168	no	yes	no	yes
		n = 81	n = 87	n = 98	n = 70
Age (years)	73 (65/86)	**69 (65/84)**	**77 (67/87)**	**71 (65/84)**	**76 (67/87)**
BMI (kg/m^2^)	27 (23/35)	27 (21/35)	27 (23/37)	**27 (21/34)**	**28 (23/38)**
Gender, m (n, (%))	78 (46.4)	40 (49.4)	38 (43.7)	**56 (57.1)**	**22 (31.4)**
Education ≤10 years (n (%))	110 (65.5)	56 (69.1)	54 (62.1)	59 (60.2)	51 (72.9)
Alcohol abstinence, yes (n (%))	23 (13.7)	8 (9.9)	15 (17.2)	**9 (9.2)**	**14 (20.0)**
Smoker or ex-smoker (n (%))	97 (57.7)	46 (56.8)	51 (58.6)	56 (57.1)	41 (58.6)
GNRI	109 (96/124)	108 (97/123)	109 (95/126)	108 (96/122)	110 (96/129)
**Health outcomes**					
Lung group, better (n (%))	92 (54.7)	47 (58.0)	45 (51.7)	58 (59.2)	34 (48.6)
Disability, yes (n (%))	70 (41.7)	**20 (24.7)**	**50 (57.5)**	–	–
Multimorbidity, yes (n (%))	87 (51.8)	–	–	**37 (37.8)**	**50 (71.4)**

Significant differences are written in bold, p≤0.05. Wilcoxon-test was used for metric variables and Chi^2^-test for categorical variables; GNRI = Geriatric Nutritional Risk Index.

#### PA and bout length

**Figure 1:**
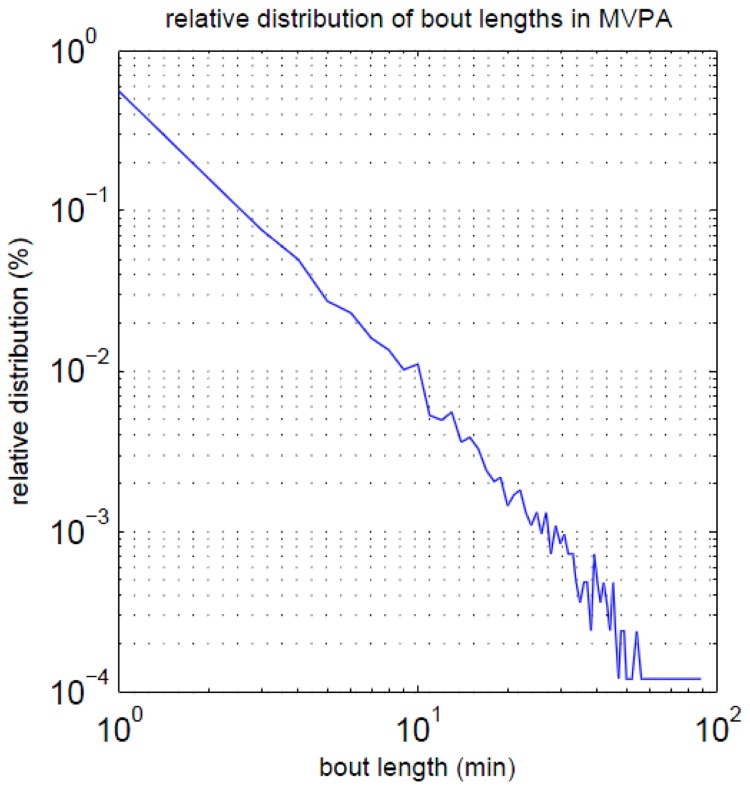
Distribution of PA bouts of moderate to vigorous physical activity.

**Figure 2:**
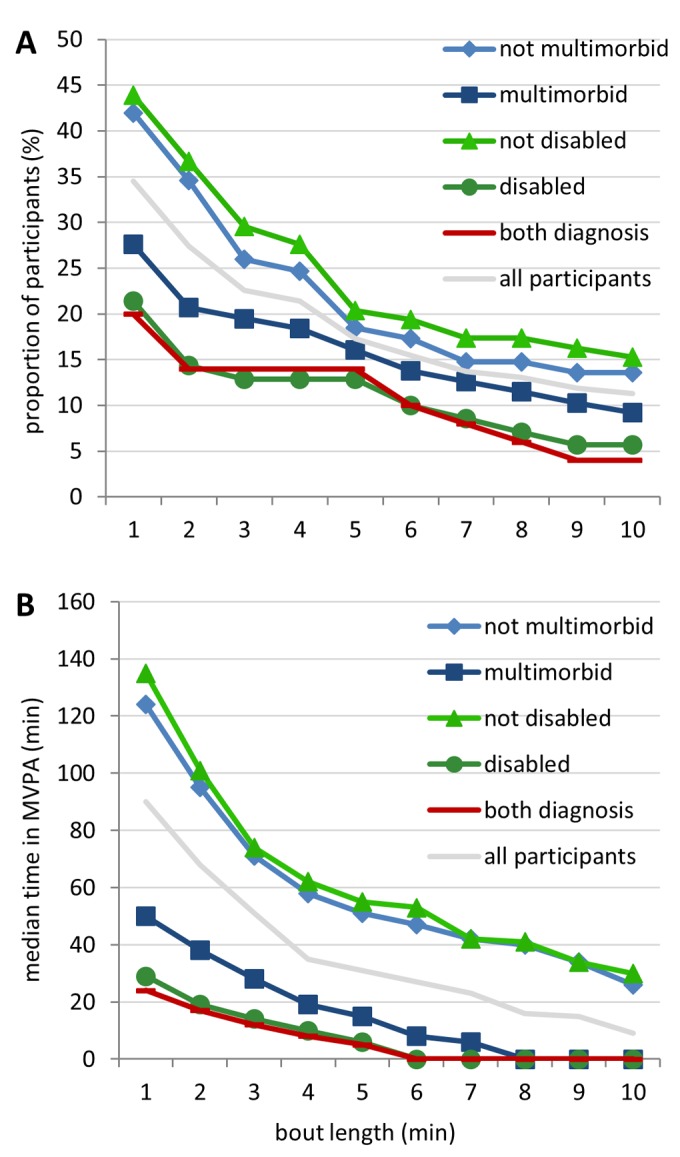
Moderate to vigorous physical activity (MVPA) in relation to different minimal bout lengths. A) Proportion of participants (%) fulfilling ≥150 min of MVPA/week vs. minimal bout length. B) Median time in MVPA per week (min). Each bout length refers to the minimal number of consecutive min in MVPA required for inclusion in the calculation of accumulated time in MVPA, i.e. if bout length is 3 then bouts of length 1 and 2 are excluded.

Overall, most of the time in MVPA (55%) was performed in bouts of one min (Figure 1). 47.6% of the participants did not achieve at least one 10-min bout. 35.7% of the participants met the current PA recommendations [11] of performing ≥150 min of MVPA, regardless of bout length, and 11.9% reached this in bouts of at least 10 min (Figure 2A). Differences in terms of MVPA between people with and without a diagnosis of disability or multimorbidity were stronger under consideration of short bouts instead of only using longer bouts, and more obvious with regard to disablity compared to multimorbidity. Furthermore, there was little difference between disabled individuals and people which were both disabled and multimorbid (Figure 2A and 2B). In addition, mean values are presented in Figure S1.

Table 2 shows the PA variables stratified by presence of disability and presence of multimorbidity. The median (5%/95%) activity per day was 221 (67/487) cpm. Lower values were observed in subjects with multimorbidity (181 (62/455) cpm) and disability (174 (57 439) cpm) while subjects without multimorbidity or disability showed significantly higher values. Overall, the participants passed a median time of 65% being sedentary, 32% in light activity and 2% in MVPA. Values of G tended to increase with decreasing intensity level: G_sedentary_ = 0.63, G_light_ = 0.48, and G_MVPA_ = 0.43 (see Table S2 for mean values). According to this, the accumulated PA time in the sedentary level was composed of a higher proportion of long bouts compared to the light or MVPA level. Multimorbid participants were generally less active. They spent 4% more of their time in the sedentary level (p<0.01), 2% less in the light level (p = 0.02) and 1% less in the MVPA level (p<0.01) compared to people who were not multimorbid. Moreover, G_sedentary_ was significantly higher in multimorbid people (p<0.01). The results for disability were similar with regard to the intensity levels, but not in terms of the PA patterns: G_light_ and G_MVPA_ were significantly lower in disabled people compared to people without diagnosis of disability (p = 0.04 and p<0.01).

**Table 2:** PA variables by mulitmorbidity and disability. Median (5%/95%).

PA variables	all	n	not multimorbid	n	multimorbid	n
Average PA (cpm)	221 (67/487)	**168**	**250 (120/487)**	**81**	**181 (62/455)**	**87**
Sedentary PA time (%)	0.65 (0.48/0.82)	168	**0.63 (0.46/0.79)**	81	**0.67 (0.51/0.84)**	87
Light PA time (%)	0.32 (0.18/0.48)	168	**0.33 (0.20/0.51)**	81	**0.31 (0.16/0.48)**	87
MVPA time (%)	0.02 (0.00/0.08)	168	**0.02 (0.00/0.08)**	81	**0.01 (0.00/0.07)**	87
G_sedentary_	0.63 (0.57/0.68)	168	**0.62 (0.57/0.68)**	81	**0.64 (0.58/0.68)**	87
G_light_	0.48 (0.37/0.55)	168	0.48 (0.38/0.55)	81	0.47 (0.37/0.55)	87
G_MVPA_	0.43 (0.00/0.66)	156	0.45 (0.00/0.67)	77	0.40 (0.00/0.65)	79
	**all**	**n**	**not disabled**	**n**	**disabled**	**n**
Average PA (cpm)	221 (67/487)	**168**	**269 (119/542)**	**98**	**174 (57/439)**	**70**
Sedentary PA time (%)	0.65 (0.48/0.82)	168	**0.64 (0.46/0.77)**	98	**0.68 (0.54/0.84)**	70
Light PA time (%)	0.32 (0.18/0.48)	168	**0.32 (0.21/0.51)**	98	**0.31 (0.16/0.45)**	70
MVPA time (%)	0.02 (0.00/0.08)	168	**0.02 (0.00/0.09)**	98	**0.01 (0.00/0.07)**	70
G_sedentary_	0.63 (0.57/0.68)	168	0.63 (0.57/0.68)	98	0.63 (0.57/0.70)	70
G_light_	0.48 (0.37/0.55)	168	**0.48 (0.40/0.55)**	98	**0.47 (0.35/0.55)**	70
G_MVPA_	0.43 (0.00/0.66)	156	**0.46 (0.13/0.68)**	96	**0.37 (0.00/0.62)**	60

Results emerged from Wilcoxon-test. Significant differences are written in bold, p≤0.05; cpm = counts per minute; G = GINI-Index; high G = mainly few long bouts are responsible for the activity pattern; low G = mainly short bouts of similar length contribute to the activity pattern.

Table 3 presents associations between the six PA variables and the prevalence of multimorbidity or disability, respectively. For the multiple regression models, each PA variable was scaled by its interquartile range (IQR). The resulting regression coefficient from IQR compares a person in the middle of the upper half of the predictor distribution to a person in the middle of the lower half of the distribution.

**Table 3:** OR and 95% CI describing the associations between PA variables and prevalent diseases.

	Model 1^a^	p	Model 2^b^	p	Model 3^c^	p
**Multimorbidity**						
Sedentary PA, IQR	**1.66 (1.14, 2.40)**	**<.01**	1.32 (0.88, 2.00)	0.18	1.11 (0.70, 1.76)	0.67
Light PA, IQR	**0.88 (0.78, 0.98)**	**0.02**	0.93 (0.82, 1.06)	0.28	0.99 (0.86, 1.14)	0.86
MVPA, IQR	**0.99 (0.99, 1.00)**	**<.01**	1.00 (0.99, 1.00)	0.15	1.00 (0.99, 1.00)	0.30
G_sedentary_, IQR	**1.48 (1.12, 1.97)**	**<.01**	1.33 (0.97, 1.82)	0.07	1.46 (1.03, 2.06)	0.03
G_light_, IQR	0.90 (0.75, 1.08)	0.25	1.00 (0.81, 1.23)	0.98	1.13 (0.88, 1.46)	0.33
G_MVPA_, IQR	1.02 (0.86, 1.21)	0.79	1.03 (0.86, 1.25)	0.73	1.07 (0.86, 1.33)	0.55
**Disability**						
Sedentary PA, IQR	**1.99 (1.33, 2.98)**	**<.01**	**1.74 (1.10, 2.75)**	**0.02**	1.52 (0.91, 2.53)	0.11
Light PA, IQR	**0.84 (0.75, 0.94)**	**<.01**	**0.86 (0.76, 0.99)**	**0.03**	0.89 (0.76, 1.03)	0.11
MVPA, IQR	**0.99 (0.98, 1.00)**	**<.01**	**0.99 (0.99, 1.00**)	**0.02**	1.00 (0.99, 1.00)	0.31
G_sedentary_, IQR	1.19 (0.90, 1.58)	0.21	1.01 (0.74, 1.38)	0.94	0.89 (0.63, 1.25)	0.50
G_light_, IQR	**0.79 (0.65, 0.95)**	**0.02**	0.85 (0.68, 1.06)	0.14	0.84 (0.65, 1.09)	0.18
G_MVPA_, IQR	0.99 (0.83, 1.18)	0.88	0.96 (0.80, 1.16)	0.68	0.96 (0.79, 1.17)	0.70

Significant estimates are written in bold, p≤0.05. P-values result from multiple logistic regression models.

a unadjusted.

b adjusted for age and sex.

c also adjusted for BMI, smoking, education, alcohol consumption, nutrition, lung function, and multimorbidity or disability.

G = GINI-Index; high G = mainly few long bouts are responsible for the activity pattern; low G = mainly short bouts of similar length contribute to the activity pattern;

#### PA and multimorbidity

The risk of multimorbidity seemed to be 1.7 times higher for an elderly person with a typical ‘high’ (75^th^ percentile) value on sedentary PA compared to a person with a typically ‘low’ (25^th^ percentile) value (model 1). In contrast, high values of light PA and MVPA showed a protective effect (OR = 0.88, p = 0.02 and OR = 0.99, p<0.01). Since the participants spent little time in MVPA and the variance of MVPA was very low, even an OR of 0.99 emerged as a statistically significant risk. High G_sedentary_ values appeared to increase the risk of multimorbidity by the factor 1.5 compared to low values. After adjustment for age and sex (model 2) all results tended into the same direction, however they were not significant, anymore. The same applied to model 3 (also adjusted for BMI, smoking, education, alcohol consumption, nutrition, lung function, and disability). Instead, higher age and presence of disability were significant predictors for multimorbidity, no matter which of the PA variables was included in the model.

#### PA and disability

The risk of disability appeared to be twice as high for participants with a typical ‘high’ value on sedentary PA compared to people with a typically ‘low’ value. In contrast, high values of light PA and MVPA showed a significant protective effect (OR = 0.84, p<0.01 and OR = 0.99, p<0.01). Due to the low variance of MVPA, even an OR of 0.99 emerged as a significant result. High G_light_ values reduced the risk of multimorbidity by 21% compared to low values. Significant associations between the three intensity levels and disability persisted, even after adjustment for age and sex (model 2). However, there were no significant relations, anymore, after controlling for further covariates (model 3). Instead, female gender and presence of multimorbidity emerged as significant risk factors for disability, no matter which of the PA variables was included in the model.

When comparing the models of the two health outcomes multimorbidity and disability, all results point in the same direction. According to model 2, the relationship between disability and PA seemed to be stronger than the relationship between multimorbidity and PA. The more variables were included in the model, the weaker became the associations between PA variables and health outcomes.

## Discussion

To the best of our knowledge, this is the first study examining the association between PA patterns and multimorbidity or disability on a sample of elderly people using accelerometry. One striking finding was that shorter bouts (e.g. 1-min bouts) of MVPA seem to provide more distinct evidence about the positive effect of MVPA with multimorbidity and disability than longer bouts (e.g. 10-min bouts). An explanation for this may be the relatively short overall time that elderly people spend in this level and especially the low proportion of long bouts. This finding is in contrast to the current PA recommendations [11] which request that PA is performed in bouts of at least 10 min (in the following called ‘10-min bout rule’) for beneficial health effects.

The majority of older adults (61.3%) failed to meet the PA recommendations [11] of 150 min of MVPA per week. When the 10-min bout rule was applied, only 11.9% fulfilled the recommendations. Differences in terms of MVPA between people with and without disability or multimorbidity were more obvious in connection with disability than with multimorbidity. Furthermore, disability seemed to be the limiting factor with regard to MVPA in people which were both disabled and multimorbid. Troiano et al. [34] reported that 7.6% of people ≥60 years met the recommendations of at least 150 min MVPA per week and according to MVPA performed in 10-min bouts only 2.4%. According to Tucker et al. [13] 8.5% of people aged 60–69 and 6.3% of people ≥70 years met the recommendations taking into account the 10-min bout rule. An explanation for the somewhat higher rates of people achieving the recommendations in our study might be that the participants had to attend the KORA study center and thus seemed to have better general health as compared to other studies: The prevalence of multimorbidity (defined as 2+ concurrent diseases) of older people widely varied (55−98%) across studies [4]. A large epidemiological study by van den Bussche et al. [35] determined a multimorbidity prevalence of 62% in Germany among people aged 65 or above and a comparable study about disability observed a prevalence of 63.6% [36] while we observed lower prevalence values, 52% of elderly people in our population were multimorbid and 42% were disabled.

There were differences between participants with and without a diagnosis of multimorbidity in terms of all intensity levels (sedentary PA, light PA, MVPA). The more time people spent actively, the lower was the risk of multimorbidity, regardless intensity of the movement. PA patterns of elderly people with and without multimorbidity only differed with regard to the PA level: people without a diagnosis seemed to get up more often. The associations were lost after adjusting for BMI, smoking, education, alcohol consumption, nutrition, lung function, and disability. Only higher age and disability showed significant associations with multimorbidity. Few large population-based health studies also investigated the relationship between PA and multimorbidity or the number of chronic diseases and reached inconsistent results: Hudon et al. [5] examined people aged 45–68 and found no relationship between multimorbidity and PA levels when age, education, income, employment, long-term limitations on activity, self-rated general health, and psychological distress were controlled for. In contrast, a study about Brazilian women aged 40 to 65 determined an increased likelihood of having two or more morbid conditions for inactive women after adjustment for sociodemographic, behavioral, clinical, and reproductive factors [6]. Likewise, two other studies reported an associations between multimorbidity and the frequency of PA (15 min, ≥12 times per month for positive effects) [37] or a mean PA score [7] in people aged ≥65 years of age after adjusting for predictor variables. However, comparisons between these studies and our results are limited because they used questionnaires to assess PA. Furthermore, the studies used different covariates, different definitions of PA (e.g. ‘active’ and ‘inactive’ vs. continuous), different definitions of multimorbidity (e.g. ‘multimorbid’ vs. ‘not multimorbid’ vs. number of chronic conditions) and they partially included other age groups. Nonetheless, the present study supports the findings by Hudon et al. [5] that there is no association between multimorbidity and PA after controlling for covariates, based on objective assessment of PA.

According to the univariate results, PA was associated with disability. The risk for disability was twice as high in people with a high proportion of time spent sedentarily compared to people with less time in this level, whereas high levels of light PA and MVPA seemed protective (OR = 0.84 and OR = 0.99). Since the participants spent little time in MVPA and the variance of MVPA was very low, the OR of 0.99 emerged as significant risk. PA patterns of elderly people with and without disability only differed in terms of light PA. A high proportion of long bouts in light activity appeared protective against disability (OR = 0.8). After adjustment of the predictor variables, there were no significant associations, anymore. Even if PA parameters do not appear to be related to disability, it is related to other variables such as gender and multimorbidity. Strong evidence from questionnaire-based literature indicates that older adults who participate in regular PA have a reduced risk of functional limitations [32], [38]. Significant results were shown for MVPA after controlling for potential predictors, whereas findings for light PA are inconsistent [39]. We found two other accelerometer-based studies which researched the relationship between PA and disability. They showed a significant association between daily PA and disability after controlling for other predictors [40], [41]. However, they did not provide information about the exact intensity levels they applied.

Neither of the mentioned studies used a disability or multimorbidity index that accounted for the severity of the functional limitation or of any disease. A previous study showed that chronic diseases were only associated with another health outcome when the severity of the disease was considered [42].

To our knowledge, the present study is the first one that examined the association between accelerometry-assessed PA levels and multimorbidity in elderly people and the only one that presents information about the relationship of PA patterns with disability and multimorbidity. Due to the objective measurement, our results are not affected by recall biases associated with self-report in older people at risk for cognitive decline.

However, since our participants had to visit the study center, a selection bias is possible which may be responsible for somewhat lower rates of multimorbid and disabled people and slightly increased MVPA. As our study was cross-sectional, we cannot assure causality for any of the associations. For greater understanding of the associations, longitudinal or intervention studies are required. Another limitation of this study is the limited validity of the cut-points applied to classify activities into intensity levels [43] in elderly people [44]. We chose the algorithm by Freedson et al. [28] because it had the highest potential to provide comparable data [14], [19], [45]. More information on this issue can be found elsewhere [22]. Furthermore, the GT3X accelerometer used in this study is likely to underestimate activities due to its inability to measure water-related activities (e.g. swimming) and to detect the types of activities performed (e.g. cycling, gymnastics, or strength trainings) [16]. Other characteristics, such as self-rated health status or psychological distress level, may have been included in the model [5]. However, we wanted this study to be focused on the relationship of PA with multimorbidity and disability and decided not to include further covariates in the analysis.

## Conclusion

In elderly people, MVPA seems to have a stronger relationship with multimorbidity and disability when shorter bouts and not only longer bouts were counted. In older people who are both disabled and multimorbid, disability seems to be the limiting factor for the performance of MVPA. From the univariate analysis presented, the following conclusions can be drawn: multimorbid people should interrupt their periods of inactivity more frequently, whereas disabled people should increase the proportion of long bouts in light activities. Again, associations between disability and PA were stronger than those between multimorbidity and PA. These conclusions should be treated with caution and are limited in their validity, since the findings were not significant after adjustment for covariates. According to multiple logistic regression analyses other variables emerged as significant predictors: higher age and disability were associated with higher risk of multimorbidity; female gender and multimorbidity were associated with higher risk of disability. Studies which take into account the severity of the disease of functional limitation could contribute to a greater understanding of the associations.

## Supporting Information

Figure S1 **Moderate to vigorous physical activity (MVPA) in relation to different minimal bout lengths.** Mean (SD) time in MVPA per week (min). Each bout length refers to the minimal number of consecutive min in MVPA required for inclusion in the calculation of accumulated time in MVPA, i.e. if bout length is 3 then bouts of length 1 and 2 are excluded.
(TIF)

Table S1 **Characteristics of the participants, stratified by multimorbidity and disability.** Mean (SD). GNRI = Geriatric Nutritional Risk Index.
(DOC)

Table S2 **PA variables by mulitmorbidity and disability.** Mean (SD). cpm = counts per minute; G = GINI-Index; high G = mainly few long bouts are responsible for the activity pattern; low G = mainly short bouts of similar length contribute to the activity pattern.
(DOC)
