# Supplementary figures and images for: Associations between Multiple Accelerometry-Assessed Physical Activity Parameters and Selected Health Outcomes in Elderly People – Results from the KORA-Age Study
Source: PLoS One. 2014 Nov 5;9(11):e111206. doi: 10.1371/journal.pone.0111206 (PMC4220984; doi:10.1371/journal.pone.0111206)

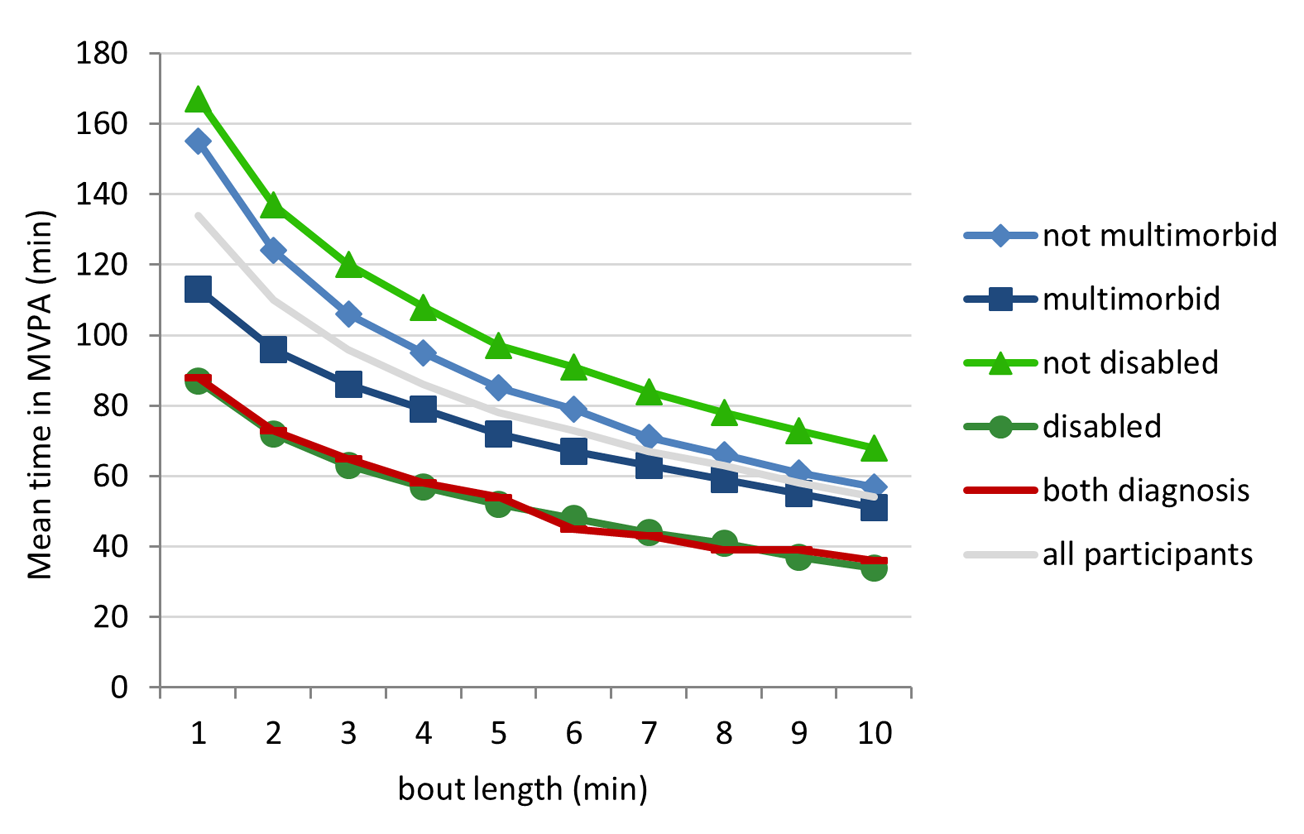

Supplement: Figure S1 — Moderate to vigorous physical activity (MVPA) in relation to different minimal bout lengths. Mean (SD) time in MVPA per week (min). Each bout length refers to the minimal number of consecutive min in MVPA required for inclusion in the calculation of accumulated time in MVPA, i.e. if bout length is 3 then bouts of length 1 and 2 are excluded. (TIF) [file pone.0111206.s001.tif]
