# Supplementary material for: Associations between Multiple Accelerometry-Assessed Physical Activity Parameters and Selected Health Outcomes in Elderly People – Results from the KORA-Age Study
Source: PLoS One. 2014 Nov 5;9(11):e111206. doi: 10.1371/journal.pone.0111206 (PMC4220984; doi:10.1371/journal.pone.0111206)
Supplement: Table S1 — Characteristics of the participants, stratified by multimorbidity and disability. Mean (SD). GNRI = Geriatric Nutritional Risk Index. (DOC) [file pone.0111206.s002.doc]

**Table S1:** Characteristics of the participants, stratified by multimorbidity and disability. Mean (SD)

|  | **all** | **multimorbidity** | | **disability** | |
| --- | --- | --- | --- | --- | --- |
|  | n=168 | **no** | **yes** | **no** | **yes** |
|  |  | n=81 | n=87 | n=98 | n=70 |
| Age (years) | 74.1 (6.5) | 71.6 (5.7) | 76.5 (6.4) | 72.5 (6.0) | 76.4 (6.7) |
| BMI (kg/m^2^) | 27.8 (4.0) | 27.2 (3.9) | 28.3 (4.1) | 27.0 (3.3) | 28.8 (4.7) |
| Gender, m (n, (%)) | 78 (46.4) | 40 (49.4) | 38 (43.7) | 56 (57.1) | 22 (31.4) |
| Education ≤10 years (n (%)) | 110 (65.5) | 56 (69.1) | 54 (62.1) | 59 (60.2) | 51 (72.9) |
| Alcohol abstinence, yes (n (%)) | 23 (13.7) | 8 (9.9) | 15 (17.2) | 9 (9.2) | 14 (20.0) |
| Smoker or ex-smoker (n (%)) | 97 (57.7) | 46 (56.8) | 51 (58.6) | 56 (57.1) | 41 (58.6) |
| GNRI | 108.9 (8.3) | 108.3 (8.0) | 109.5 (8.7) | 108.0 (7.4) | 110.2 (9.3) |
| **Health outcomes** |  |  |  |  |  |
| Lung group, better (n (%)) | 92 (54.7) | 47 (58.0) | 45 (51.7) | 58 (59.2) | 34 (48.6) |
| Disability, yes (n (%)) | 70 (41.7) | 20 (24.7) | 50 (57.5) | - | - |
| Multimorbidity, yes (n (%)) | 87 (51.8) | - | - | 37 (37.8) | 50 (71.4) |

GNRI=Geriatric Nutritional Risk Index
