# Supplementary material for: Associations between Multiple Accelerometry-Assessed Physical Activity Parameters and Selected Health Outcomes in Elderly People – Results from the KORA-Age Study
Source: PLoS One. 2014 Nov 5;9(11):e111206. doi: 10.1371/journal.pone.0111206 (PMC4220984; doi:10.1371/journal.pone.0111206)
Supplement: Table S2 — PA variables by mulitmorbidity and disability. Mean (SD). cpm = counts per minute; G = GINI-Index; high G = mainly few long bouts are responsible for the activity pattern; low G = mainly short bouts of similar length contribute to the activity pattern. (DOC) [file pone.0111206.s003.doc]

**Table S2:** PA variables by mulitmorbidity and disability. Mean (SD)

| **PA variables** | **all** | **n** | **not multimorbid** | **n** | **multimorbid** | **n** |
| --- | --- | --- | --- | --- | --- | --- |
| Average PA (cpm) | 243 (129) | 168 | 269 (122) | 81 | 218 (131) | 87 |
| Sedentay PA time (%) | 0.65 (0.10) | 168 | 0.63 (0.10) | 81 | 0.67 (0.09) | 87 |
| Light PA time (%) | 0.32 (0.09) | 168 | 0.34 (0.09) | 81 | 0.31 (0.08) | 87 |
| MVPA time (%) | 0.02 (0.03) | 168 | 0.03 (0.02) | 81 | 0.02 (0.03) | 87 |
| G_sedentary_ | 0.63 (0.04) | 168 | 0.62 (0.03) | 81 | 0.64 (0.03) | 87 |
| G_light_ | 0.40 (0.05) | 168 | 0.43 (0.05) | 81 | 0.36 (0.05) | 87 |
| G_MVPA_ | 0.43 (0.20) | 156 | 0.45 (0.18) | 77 | 0.40 (0.21) | 79 |
|  | **all** | **n** | **not disabled** | **n** | **disabled** | n |
| Average PA (cpm) | 243 (129) | 168 | 277 (129) | 98 | 195 (114) | 70 |
| Sedentay PA time (%) | 0.65 (0.10) | 168 | 0.63 (0.10) | 98 | 0.68 (0.09) | 70 |
| Light PA time (%) | 0.32 (0.09) | 168 | 0.34 (0.09) | 98 | 0.30 (0.09) | 70 |
| MVPA time (%) | 0.02 (0.03) | 168 | 0.03 (0.03) | 98 | 0.02 (0.03) | 70 |
| G_sedentary_ | 0.63 (0.04) | 168 | 0.63 (0.03) | 98 | 0.63 (0.04) | 70 |
| G_light_ | 0.47 (0.05) | 168 | 0.48 (0.05) | 98 | 0.46 (0.06) | 70 |
| G_MVPA_ | 0.40 (0.20) | 156 | 0.44 (0.17) | 96 | 0.32 (0.21) | 60 |

cpm=counts per minute; G=GINI-Index; high G=mainly few long bouts are responsible for the activity pattern; low G=mainly short bouts of similar length contribute to the activity pattern
